# ‘*Candidatus* Phytoplasma phoenicium’ associated with almond witches’-broom disease: from draft genome to genetic diversity among strain populations

**DOI:** 10.1186/s12866-015-0487-4

**Published:** 2015-07-30

**Authors:** Fabio Quaglino, Michael Kube, Maan Jawhari, Yusuf Abou-Jawdah, Christin Siewert, Elia Choueiri, Hana Sobh, Paola Casati, Rosemarie Tedeschi, Marina Molino Lova, Alberto Alma, Piero Attilio Bianco

**Affiliations:** Department of Agricultural and Environmental Sciences - Production, Landscape, Agroenergy, Università degli Studi di Milano, via Celoria 2, 20133 Milan, Italy; Division Phytomedicine, Thaer-Institute, Humboldt-Universität zu Berlin, Berlin, Germany; Faculty of Agricultural and Food Sciences, American University of Beirut, Beirut, Lebanon; Department of Plant Protection, Lebanese Agricultural Research Institute, Tal Amara, Lebanon; Department of Agricultural, Forest and Food Sciences, Università degli Studi di Torino, Grugliasco (TO), Italy; AVSI Foundation, Jounieh, Ghadir Lebanon

**Keywords:** Phytoplasma, Parasitism, Vector, Integral membrane protein, BI-1

## Abstract

**Background:**

Almond witches’-broom (AlmWB), a devastating disease of almond, peach and nectarine in Lebanon, is associated with ‘*Candidatus* Phytoplasma phoenicium’. In the present study, we generated a draft genome sequence of ‘*Ca*. P. phoenicium’ strain SA213, representative of phytoplasma strain populations from different host plants, and determined the genetic diversity among phytoplasma strain populations by phylogenetic analyses of *16S rRNA*, *groEL*, *tufB* and *inmp* gene sequences.

**Results:**

Sequence-based typing and phylogenetic analysis of the gene *inmp*, coding an integral membrane protein, distinguished AlmWB-associated phytoplasma strains originating from diverse host plants, whereas their *16S rRNA*, *tufB* and *groEL* genes shared 100 % sequence identity. Moreover, dN/dS analysis indicated positive selection acting on *inmp* gene. Additionally, the analysis of ‘*Ca*. P. phoenicium’ draft genome revealed the presence of integral membrane proteins and effector-like proteins and potential candidates for interaction with hosts. One of the integral membrane proteins was predicted as BI-1, an inhibitor of apoptosis-promoting Bax factor. Bioinformatics analyses revealed the presence of putative BI-1 in draft and complete genomes of other ‘*Ca*. Phytoplasma’ species.

**Conclusion:**

The genetic diversity within ‘*Ca*. P. phoenicium’ strain populations in Lebanon suggested that AlmWB disease could be associated with phytoplasma strains derived from the adaptation of an original strain to diverse hosts. Moreover, the identification of a putative inhibitor of apoptosis-promoting Bax factor (BI-1) in ‘*Ca*. P. phoenicium’ draft genome and within genomes of other ‘*Ca*. Phytoplasma’ species suggested its potential role as a phytoplasma fitness-increasing factor by modification of the host-defense response.

**Electronic supplementary material:**

The online version of this article (doi:10.1186/s12866-015-0487-4) contains supplementary material, which is available to authorized users.

## Background

Phytoplasmas are bacterial plant pathogens that cause economically relevant yield losses in several low- and high- value annual and perennial crops worldwide, including fruit and woody trees [1, 2]. They are transmitted plant-to-plant by phloem feeders of the order Hemiptera, mostly leafhoppers (Cicadellidae), planthoppers (Fulgoroidea) and psyllids (Psyllidae) [3]. Phytoplasmas are classified into the class *Mollicutes,* which includes bacteria with single membrane that have diverged from a Gram-positive ancestor [4, 5]. Based on molecular and other biological features phytoplasma strains have been classified into 37 species within the provisional genus ‘*Candidatus* Phytoplasma’ [6, 7] and taxonomic groupings have also been delimited according to the DNA sequence coding for their 16S ribosomal RNA [8].

Phytoplasmas of taxonomic group 16SrIX (pigeon pea witches’-broom group) are associated with diseases affecting crop and wild plants in different geographic areas worldwide [9–12]. ‘*Candidatus* Phytoplasma phoenicium’, taxonomic subgroup 16SrIX-B [13, 14], also designated as 16SrIX-D [15, 16], and its genetic variants [16] are the etiological agents of a lethal devastating disease of almond trees (almond witches’-broom, AlmWB) in Lebanon and in Iran [10, 13, 17, 18]. A similar disease, inducing almond broomings, was reported in Iran [10] in association with phytoplasmas close to those responsible for *Knautia arvensis* phyllody (KAP), subgroup 16SrIX-C [18]. Furthermore, almond trees showing different symptoms, such as small and yellow leaves, were found infected by ‘*Ca*. P. aurantifolia’, ‘*Ca*. P. solani’ and ‘*Ca*. P. trifolii’ in Iran [19]. AlmWB was also identified on peach (*P. persica*) and nectarine (*P. persica* var. *nucipersica*) in southern Lebanon [20] and on GF-677 (*P. amygdalus* x *P. persica*) in Iran [21].

The most characteristic symptoms caused by AlmWB on almond trees are (i) shoot proliferation on the main trunk with the appearance of a witches’-broom, (ii) the perpendicular development of many axillary buds on the branches, with smaller and yellowish leaves, (iii) the general decline of the tree with final dieback. A total loss of production happens 1–2 years after the initial appearance of the symptoms [13]. In the case of peach and nectarine trees, the first symptom observed is early flowering (15 to 20 days earlier than normal), followed by the earlier development of all the buds of the infected branches. In addition, phyllody at the flowering period and serrate, slim, light green leaves and witches’-brooms developing from the trunk and the crown of the trees several months after are observed [20].

Due to complex ecology of ‘*Ca*. P. phoenicium’, associated with diverse crops, in which it induces variations in symptoms, it is necessary to evaluate the genetic diversity among AlmWB phytoplasma populations in order to determine phytoplasma strain-specific molecular markers. In previous studies, genetic heterogeneity among phytoplasma strain populations was investigated by multilocus sequence typing analyses [22–24]. Recently, Lee and colleagues [14] found that ‘*Ca*. P. phoenicium’ strains from Lebanon shared identical nucleotide sequences of the genes *rplV*-*rpsC* and *secY*, distinct from phytoplasmas belonging to other 16SrIX taxonomic subgroups. Such findings highlighted that more variable genes should be investigated to determine the diversity among ‘*Ca*. P. phoenicium’ strains. In previous studies, draft or full length genome sequencing allowed not only the acquisition of key information on phytoplasma metabolism and mechanisms of interaction with hosts [25–33], but also the identification of hyper-variable genes suitable for distinguishing closely related strains of ‘*Ca*. P. solani’ (genes *vmp* and *stamp*) [34–36] and ‘*Ca*. P. mali’ (gene *hflB*) [27, 37].

In the present study, we obtained the draft genome sequence of a ‘*Ca*. P. phoenicium’ strain SA213 identified in almond from Lebanon. Different house-keeping and variable genes were selected in order to determine genetic variability among ‘*Ca*. P. phoenicium’ strain populations by sequencing and phylogenetic analyses. The results obtained reveal useful information about the complexity of ‘*Ca*. P. phoenicium’ population structure, and highlight intriguing preliminary insights into the possible interactions with hosts of this phytoplasma.

## Methods

### Sample collection and DNA extraction

During the spring season of 2012, leaves and shoots from plants showing typical AlmWB symptoms were collected. Specifically, samples were obtained from almond, peach, and nectarine in North (Lat 34.207880, Long 35.677113) and South Lebanon (Lat 33.278748, Long 35.554823), while almond shoots only were sampled in the Bekaa valley (Lat 34.015622, Long 36.176262) (Table 1). After surface disinfection, the phloem shavings or the leaf veins (about 100 mg per sample) were placed in 1.5 ml tubes, immersed immediately in liquid nitrogen and ground using a pestle attached to an electrical drill. The small scale CTAB protocol was used to extract total nucleic acids as previously described [13]. The nucleic acid pellet was washed with 75 % ethanol, air-dried, suspended in 100 μl of sterile water, and maintained at −80 °C until use.

**Table 1:** Host, sampling location and accession numbers of *16S rRNA*, *tufB*, *groEL* and *inmp* gene nucleotide sequences of analyzed ‘*Ca*. P. phoenicium’ strains

Strain	Host	Locality	Accession No.			
			*16S rRNA* ^a^	*tufB* ^b^	*groEL* ^c^	*inmp* ^d^
A48	*Prunus dulcis* (Mill.) D.A.Webb	Bekaa				
A49	*Prunus dulcis* (Mill.) D.A.Webb					
A50	*Prunus dulcis* (Mill.) D.A.Webb					
A51	*Prunus dulcis* (Mill.) D.A.Webb					
A60	*Prunus dulcis* (Mill.) D.A.Webb					
A65	*Prunus dulcis* (Mill.) D.A.Webb					
NA202-1	*Prunus dulcis* (Mill.) D.A.Webb	North				
NA206-1	*Prunus dulcis* (Mill.) D.A.Webb					
NA223-1	*Prunus dulcis* (Mill.) D.A.Webb					KM275495
NN204-1	*Prunus persica* var. *nucipersica*					
NN209-2	*Prunus persica* var. *nucipersica*					KM275499
NP207-1	*Prunus persica* (L.) Batsch					KM275496
SA202	*Prunus dulcis* (Mill.) D.A.Webb	South				
SA213	*Prunus dulcis* (Mill.) D.A.Webb		KM275491	KM275492	KM275493	KM275494
SA214	*Prunus dulcis* (Mill.) D.A.Webb					
SA216	*Prunus dulcis* (Mill.) D.A.Webb					
SN205	*Prunus persica* var. *nucipersica*					
SN209	*Prunus persica* var. *nucipersica*					
SP202	*Prunus persica* (L.) Batsch					KM275498
SP232	*Prunus persica* (L.) Batsch					KM275497

^a^ *16S rRNA* gene nucleotide sequences of other AlmWB phytoplasma strains are identical to the sequence of the strain SA213 (KM275491)

^b^ *tufB* gene nucleotide sequences of other AlmWB phytoplasma strains are identical to the sequence of the strain SA213 (KM275492) ^*c*^ *groEL* gene nucleotide sequences of other AlmWB phytoplasma strains are identical to the sequence of the strain SA213 (KM275493)

^d^ *inmp* gene nucleotide sequences of other AlmWB phytoplasma strains are identical to the sequence of the strain SA213 (KM275494)

### Phytoplasma identification by 16S rDNA amplification and sequence analyses

Total nucleic acids from the plants under investigation were employed as templates in PCR reactions amplifying the phytoplasma *16S rRNA* gene. Reactions were carried out using the universal primer pair P1/P7 [38, 39] followed by nested PCR using primer pair R16F2n/R16R2 [40], able to amplify partial 16S rDNA sequences of the known species inside the genus ‘*Ca*. Phytoplasma’. DNAs extracted from Madagascar periwinkle [*Catharanthus roseus* (L.) G. Don] plants infected by phytoplasma strains EY1 (‘*Ca.* P. ulmi’, subgroup 16SrV-A), STOL (‘*Ca.* P. solani’, subgroup 16SrXII-A), and AY1 (‘*Ca.* P. asteris’, subgroup 16SrI-B) served as reference controls. DNA from healthy periwinkle and reaction mixture devoid of DNA template were used as negative controls. PCRs were performed in an automated thermal cycler (Mastercycler gradient, Eppendorf, Hamburg, Germany). The presence of PCR amplicons was verified by electrophoresis through 1 % agarose gel.

Amplicons from nested PCRs were sequenced to achieve at least 4X sequence coverage per base position. DNA sequencing was performed in an ABI PRISM 377 automated DNA sequencer (Applied Biosystems, Carlsbad CA, USA) by a commercial service (Primm, Milan, Italy). Nucleotide sequence data were assembled by employing the CAP3 assembler module of the Bioedit software, version 7.2.5 [41]. Sequences were compared with the GenBank database using the software BlastN (http://www.ncbi.nim.nih.gov/BLAST/). Affiliation of identified phytoplasmas to taxonomic 16Sr group/subgroup was determined by *in silico* RFLP analyses of F2n/R2 amplicons carried out using the software iPhyClassifier (http://plantpathology.ba.ars.usda.gov/cgi-bin/resource/iphyclassifier.cgi) [8].

Phytoplasma *16S rRNA* gene sequences from this study (Table 1) and from GenBank were used to construct phylogenetic trees. Minimum evolution analysis was carried out using the Neighbor-Joining method and bootstrap replicated 1000 times using the software MEGA5 (http://www.megasoft-ware.net/index.html) [42].

### Genome sequencing, assembling and annotation

The ‘*Ca*. P. phoenicium’ strain SA213, identified in almond in South Lebanon, was selected as representative of phytoplasma strain populations examined in the present study for the genome sequencing. Five micrograms of DNA, extracted from phloem tissue of symptomatic shoots as described above, was used for library preparation to Illumina sequencing carried out by a commercial sequencing service (Institute of Applied Genomics, IGA, Udine, Italy). Barcoded libraries were prepared using the NEBnext DNA sample prep Kit (New England Biolabs, Ipswich, MS, USA) according to the manufacturer’s instructions. Libraries were sequenced on a MiSeq Sequencing System (Illumina, San Diego CA, USA) in a 150-base single read multiplex run.

Quality trimming of reads, mapping and *de novo* assembly was performed in CLC Genomics Workbench 6.0.2 (http://www.clcbio.com/) applying standard parameters. Nucleotide entries for *Acholeplasmataceae* deposited in GenBank (2013-01-11) were downloaded, imported in CLC Genomics Workbench and used as reference for read mapping. Reads assigned to *Acholeplasmataceae* by this approach were selected for *de novo* assembly (positive read selection). The minimal size for contiguous sequences (contigs) was set to 1000 b.

Contigs were compared via BLASTX [43] against NRPROT database (ftp://ftp.ncbi.nlm.nih.gov/blast/db/). Contigs and BLASTX data were uploaded in MEGAN (MEta Genome ANalyzer) [44] handling contigs as reads and applying a minimal support level of one and low complexity filter off. All sequences with an assignment to the phylum *Tenericutes* were selected for initial analysis but re-evaluated during the annotation with respect to an unambiguous assignment to the phytoplasma clade. The draft genome was analyzed by the automated annotation pipeline RAST [45] and manually curated in Artemis [46]. Functional protein domains of all predicted proteins were identified by InterProScan 4 [47]. Transmembrane topology and signal peptides in protein sequences from annotated genes were predicted by Phobius [48] providing information on cell localizations of proteins.

To estimate the completeness of ‘*Ca.* P. phoenicium’ draft genome, the percentage of AlmWB phytoplasma proteins included in the core-genome of ‘*Ca*. Phytoplasma’ [49] was calculated. Therefore, the ‘*Ca*. Phytoplasma’ core-genome (294 proteins) and the 333 ‘*Ca.* P. phoenicium’ proteins were used for a PanOCT analysis [50] with the standard parameters of PanOCT (identity of 20 % and e-value cut-off below 1e-05).

To analyze the genetic repertoire of ‘*Ca.* P. phoenicium’ draft genome, tblastn analyses (low complexity filter off, e-15, minimal identity 25 %) were carried out to compare how many genes (coding sequences), identified in draft genome of AlmWB phytoplasma, are present also in the complete or draft genome of other phytoplasmas available in GenBank.

### Multilocus sequence typing (MLST) analyses on ‘*Ca*. P. phoenicium’ strains

Based on a draft genome sequence of ‘*Ca*. P. phoenicium’ strain SA213, genes *tufB* (translational elongation factor Tu, EF-Tu), *groEL* (chaperonine GroEL), and *inmp* (integral membrane protein) were selected for investigating the genetic diversity among ‘*Ca*. P. phoenicium’ strain populations (Table 1) by MLST analyses. For each gene, two primer pairs were designed for carrying out nested PCR reactions (Table 2). Reaction mixture contained 1.5 mM MgCl_2_, 0.4 μM of each primer, and 0.2 mM of each dNTP. Reaction conditions were 5 min at 94 °C, 35 cycles including 1 min at 94 °C, 1 min at 50 °C (55 °C in nested PCRs), 2 min at 72 °C, and 10 min at 72 °C. DNAs extracted from periwinkle plants infected by phytoplasma strains EY1, STOL and AY1, from almond infected by ‘*Ca*. P. phoenicium’ strain SA213 (subgroup 16SrIX-B), and from *Picris eichioides* infected by phytoplasma strain PEY (*Picris eichioides* yellows phytoplasma, subgroup 16SrIX-C) served as reference controls. DNA from healthy periwinkle and reaction mixture without DNA template were used as negative controls. PCR reactions and electrophoretic analyses were performed as mentioned above.

**Table 2:** Primer pairs designed for amplifying *tufB*, *groEL* and *inmp* gene nucleotide sequences in direct (d) and nested (n) PCR reactions

Gene	Primer	Sequence (5′-3′)	Amplicon size (nt)
*tufB*	fusAF1 (d)	ATCGTGGTAATGCGATTGTGG	1431
	tufBR1 (d)	ACAGAACCAGCTCCAACAGTACGTCC	
	fusAF2 (n)	TGGTTATGCAACCACTTTACGTTC	1320
	tufBR2 (n)	TAGTGCAATAGGATGAATTAAAGTCAC	
*groEL*	groELF1 (d)	TGATAATGCAGGCGACGGAACTAC	1336
	groELR1 (d)	TCACAGCCACTACGGCAGCACCAGCTG	
	groELF2 (n)	ACTACTACAGCTACTGTATTAGCAC	1284
	groELR2 (n)	TAGATGCTGCAATAGAAGAAGCATTG	
*inmp*	inmpF1 (d)	AGTAATTAATTTTCAATATTGGACTG	668
	inmpR1 (d)	TCACATCATCCTCATTCATTTTTGAAGC	
	inmpF2 (n)	AGAAATCTTATCAGTGGTATCAGTC	413
	inmpR2 (n)	TCTTTATCTATTGTTTTATATGCCAC	

Amplicons of *tufB*, *groEL* and *inmp* genes from nested PCRs were sequenced, assembled, compared with the GenBank database, and used to construct phylogenetic trees as described above for *16S rRNA* gene. *TufB*, *groEL* and *inmp* gene nucleotide sequences of representative ‘*Ca.* P. phoenicium’ strains were deposited in the GenBank database (Table 1).

Analysis of synonymous and non-synonymous substitutions per site and codon-based test of positive selection (dN/dS) were carried out using MEGA5 [42] for genes showing nucleotide variability among AlmWB phytoplasma strains examined.

### Nucleotide sequences

This Whole Genome Shotgun project has been deposited at DDBJ/EMBL/GenBank under the accession JPSQ00000000. The version described in this paper is version JPSQ01000000. Nucleotide sequences of genes *16S rRNA*, *tufB*, *groEL*, *inmp* and *BI-1* of representative ‘*Ca.* P. phoenicium’ strains identified in the present study were deposited in the GenBank database at accession numbers KM275491 to KM275499, and KP640614.

## Results and Discussion

### 16S rDNA-based identification and phylogeny of ‘*Ca*. P. phoenicium’ in Lebanon

**Fig. 1:**
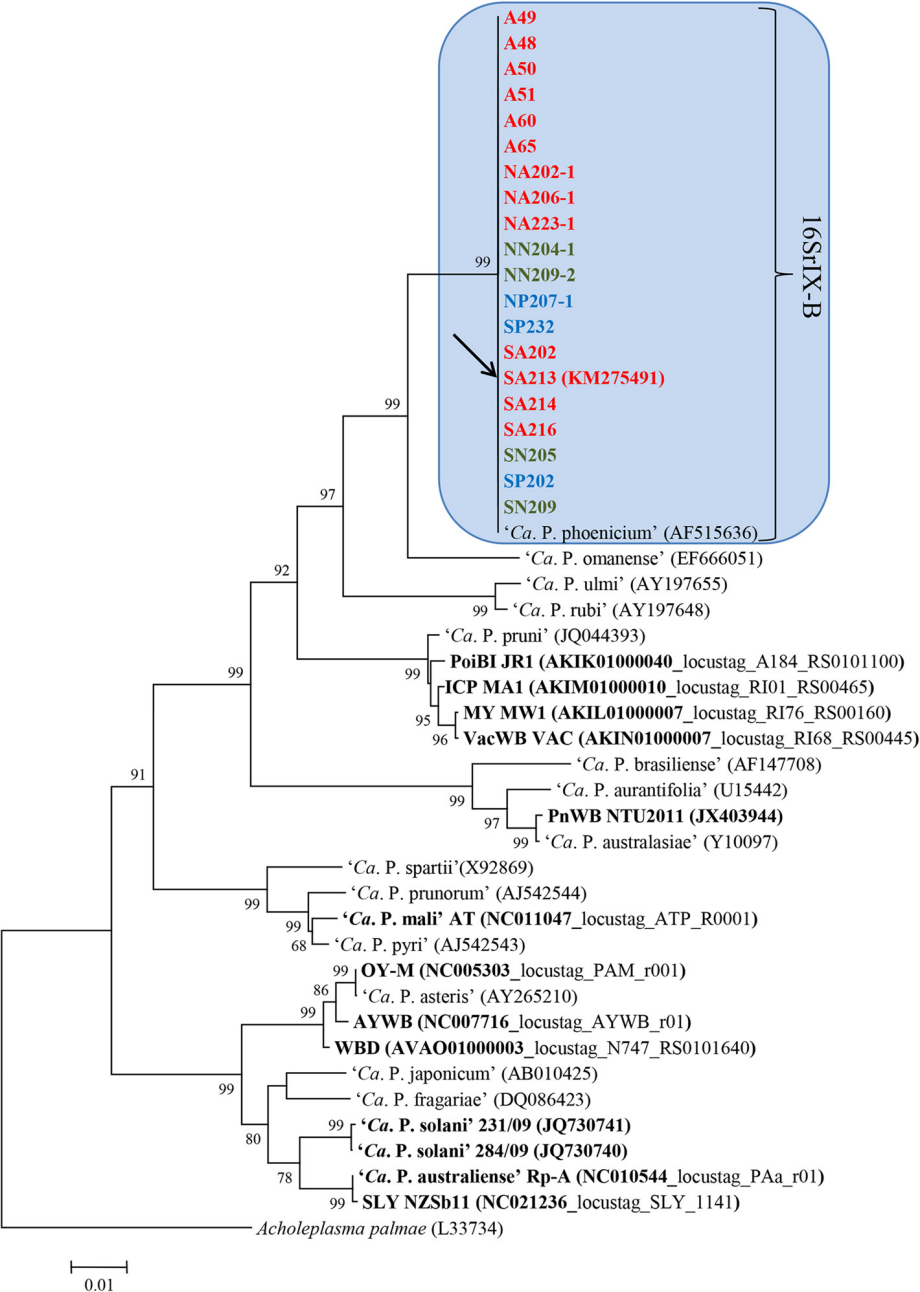
Phylogenetic tree inferred from ‘*Ca*. P. phoenicium’ strain nucleotide sequences of gene *16S rRNA*. Minimum evolution analysis was carried out using the neighbor-joining method and bootstrap replicated 1,000 times. List of ‘*Ca*. P. phoenicium’ strains is reported in Table 1. ‘*Ca*. P. phoenicium’ strains identified in almond, peach and nectarine are written in bold red, blue and green, respectively. Names of other phytoplasmas included in the phylogenetic analysis are presented on the tree image. The GenBank accession number of each sequence is given in parenthesis. Phytoplasmas, among those whose genome was partially or fully sequenced, are indicated in bold characters: PoiBI JR1, Poinsettia branch-inducing phytoplasma strain JR1; ICP MA1, Italian clover phyllody phytoplasma strain MA1; MY MW1, Milkweed yellows phytoplasma strain MW1; VacWB VAC, Vaccinium witches’-broom phytoplasma strain VAC; PnWB NTU2011, Peanut witches’-broom phytoplasma strain NTU2011; OY-M, Onion yellows phytoplasma strain OY-M; AYWB, Aster yellows witches’-broom phytoplasma strain AYWB; WBD, Wheat blue dwarf phytoplasma; SLY NZSb11, Strawberry lethal yellows phytoplasma strain NZSb11

Nested PCRs, carried out with primer pair F2n/R2, revealed the presence of phytoplasmas in all tested plants (almond, peach, and nectarine) showing AlmWB typical symptoms. PCR reliability was proven by DNA amplification from phytoplasma reference strains EY1, STOL, and AY1; no amplification was obtained from DNA of healthy plants and from the reaction mix devoid of DNA. BlastN analysis demonstrated that such phytoplasmas share 100 % sequence identity with the reference strain of the species ‘*Ca*. P. phoenicium’ (acc. no. AF515636) [10]. Sequence alignment and sequence identity calculation highlighted that ‘*Ca*. P. phoenicium’ strains, identified in distinct host plants from diverse geographic regions of Lebanon, carried identical 16S rDNA sequences (sequence identity 100 %). *In silico* RFLP (data not shown) and phylogenetic analysis (Fig. 1) confirmed the affiliation of these phytoplasma strains to the species ‘*Ca*. P. phoenicium’, taxonomic subgroup 16SrIX-B [14, 51]. This result highlighted the strict association between AlmWB symptoms on almond, peach, and nectarine plants and the infection by ‘*Ca*. P. phoenicium’ strains belonging to taxonomic subgroup 16SrIX-B and its genetic variants, reported in previous studies [16, 51]. Due to the complete 16S rDNA identity of ‘*Ca*. P. phoenicium’ strain populations, the strain SA213, identified in almond in South Lebanon, was selected as representative strain of the phytoplasma populations for genome sequencing.

### Draft genome overview

Illumina sequencing was performed for libraries resulting in 12,966,415 quality-passed short-reads with an average length of 145 bases (1,875 Tb read data in total). Assembly of 307,478 positive-selected reads resulted in 198 contigs with an average length of 2,054 bp comprising a total contig length of 406,850 bp. 78 contigs (39 %) with a 38-fold sequencing coverage could be assigned to the taxon ‘*Ca*. Phytoplasma’ using the MEGAN approach [44]. Phytoplasma assigned contigs (354 kbp, GC percentage 26 %) included 333 protein coding sequences (average length 871 bp per deduced protein), 3 *tRNA* and one *16SrRNA* gene (Additional file 1: Table S1). An estimation of the covered gene content of ‘*Ca.* P. phoenicium’ draft genome was performed by comparison to the phytoplasmas protein coding gene core set [49]. Even obtained data evidenced that ‘*Ca.* P. phoenicium’ draft genome is far away from being complete [49], it can be shown that 81 % (238 protein coding genes) of the core set protein coding genes are also included in the ‘*Ca.* P. phoenicium’ draft sequence (Additional file 2: Table S2). Moreover, tblastn analyses evidenced the presence of 34 proteins (32 described as ‘hypothetical protein’) predicted to be unique for ‘*Ca.* P. phoenicium’ (Additional file 3: Tabel S3). Based on the presence of transmembrane domain and/or secretion signal peptide, identified by Phobius analysis, 19 and 14 AlmWB phytoplasma-unique proteins were predicted to be membrane and cytosolic proteins, respectively. One protein was predicted to be secreted (Additional file 3: Table S3). Due to the phytoplasma lifestyle (obligate intracellular parasitism), it should be interesting to carry out additional studies to determine the role and activity of predicted membrane and secreted proteins identified only in AlmWB phytoplasma. Obtained data should be useful to improve the knowledge of mechanisms of interaction between ‘*Ca*. P. phoenicium’ and its host(s).

Tblastn analysis results evidenced the best number of shared proteins between AlmWB phytoplasma and strains of the species ‘*Ca*. P. pruni’ (taxonomic group 16SrIII) underlying their close relationship, also evidenced by phylogenetic analysis (Fig. 1, Additional file 3: Table S3).

### Multiple gene typing of AlmWB phytoplasma strain populations

Based on draft genome sequence, genes *tufB*, *groEL*, and *inmp* were selected for investigating the genetic diversity among ‘*Ca*. P. phoenicium’ strain populations by MLST analyses. In previous studies, nucleotide sequence analyses of *tufB* and *groEL* housekeeping genes allowed to distinguish closely related phytoplasma strains of the species ‘*Ca*. P. solani’ and ‘*Ca*. P. asteris’, showing also the relationship between strain-specific molecular markers and biological features of the phytoplasma strain(s) [52, 53]. As distinction resolution power among closely related phytoplasma strains was improved by the molecular characterization of hyper-variable genes coding membrane proteins [34–36], *inmp* gene, coding an integral membrane protein predicted to be unique for AlmWB phytoplasma, was chosen to determine the genetic diversity among ‘*Ca*. P. phoenicium’ strain populations in Lebanon. Amplicons of *tufB*, *groEL*, and *inmp* genes were obtained by nested PCRs from the 20 AlmWB phytoplasma-infected plants, examined in the present study, including the almond plant infected by ‘*Ca*. P. phoenicium’ strain SA213 (subgroup 16SrIX-B). No amplification could be generated from plants infected by other phytoplasmas of 16Sr groups I, V, XII and of subgroup IX-C, and from reaction mixtures devoid of DNA. This result demonstrated the 16SrIX-B subgroup-specificity of the primer pairs designed in the present study.

Multiple sequence analyses and sequence identity determination showed that all AlmWB phytoplasma strains shared 100 % identity of *tufB* and *groEL* gene sequence fragments. Alternatively, 15 AlmWB phytoplasma strains (including the strain SA213, selected for the draft genome sequencing) shared identical *inmp* gene nucleotide sequences, while 5 AlmWB phytoplasma strains exhibited a nucleotide sequence identity from 93.5 to 99.7 % with respect to the SA213 strain sequence. Interestingly, three strains exhibiting lower identities (from 93.5 to 98.3 %) were isolated from peach plants (Additional file 4: Table S4).

**Fig. 2:**
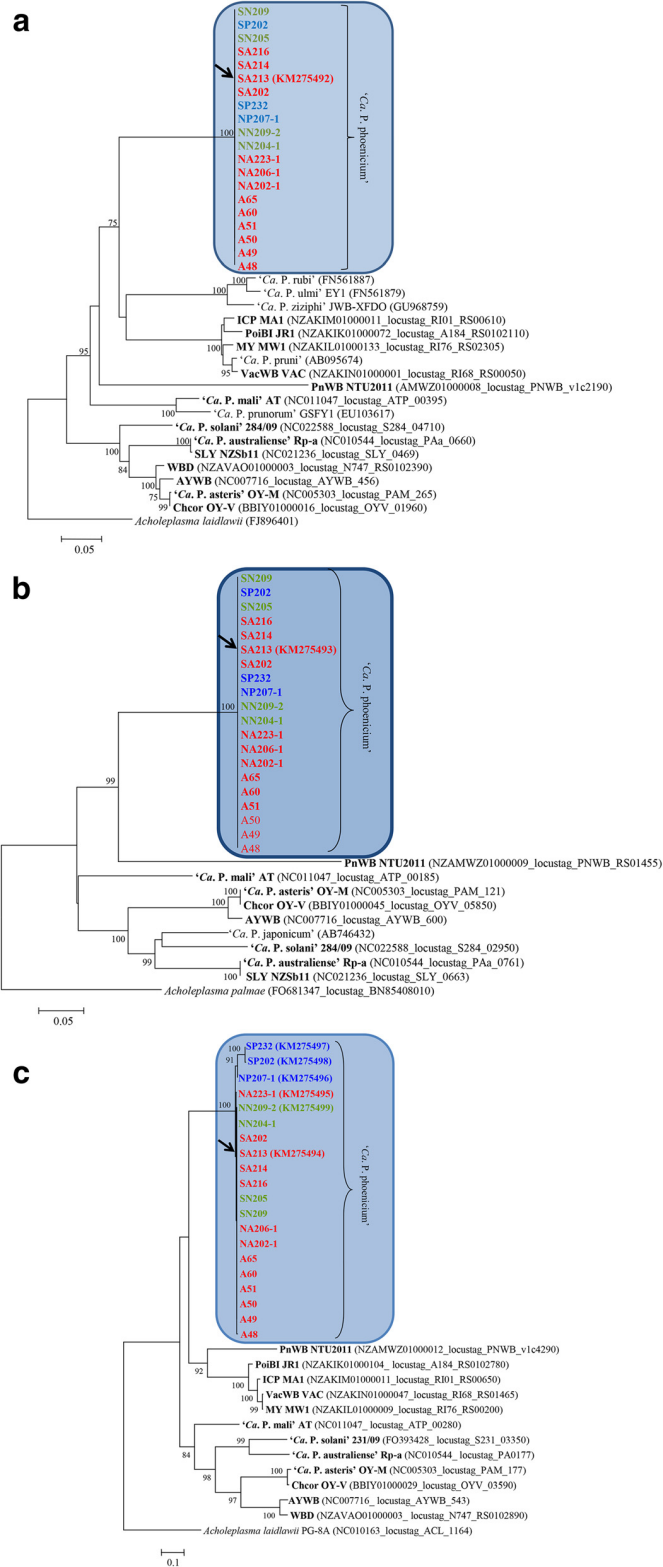
Phylogenetic trees inferred from ‘*Ca*. P. phoenicium’ strain nucleotide sequences of genes *tufB* (**a**), *groEL* (**b**), and *inmp* (**c**). Minimum evolution analysis was carried out using the neighbor-joining method and bootstrap replicated 1,000 times. List of ‘*Ca*. P. phoenicium’ strains is reported in Table 1. ‘*Ca*. P. phoenicium’ strains identified in almond, peach and nectarine are written in bold red, blue and green color, respectively. Names of other phytoplasmas included in phylogenetic analysis are written on the tree image. Acronyms of phytoplasma names are in the legend of Fig. 1. GenBank accession number of each sequence is given in parenthesis

Phylogenetic analyses confirmed the presence of one cluster based on *tufB* and *groEL* genes, and of one main cluster including a subcluster (peach infecting strains) based on the *inmp* gene (Fig. 2a, b, c, respectively). Nucleotide sequence analyses revealed that AlmWB phytoplasma strain SA213 is representative of the ‘*Ca*. P. phoenicium’ strain populations in Lebanon. Based on phylogenetic trees, PnWB phytoplasma, ‘*Ca*. P. mali’ and four phytoplasmas of group 16SrIII (Poinsettia branch-inducing phytoplasma, Milkweed yellows phytoplasma, Italian clover phyllody phytoplasma, and Vaccinium witches broom phytoplasma) show the closest relationship to ‘*Ca*. P. phoenicium’ among the phytoplasmas whose draft or complete genome has already been published. Evidence for a close relationship with PnWB phytoplasma and group 16SrIII phytoplasmas is also supported by common genome features, as evidenced by tblastn analysis (Additional file 3: Table S3).

**Fig. 3:**
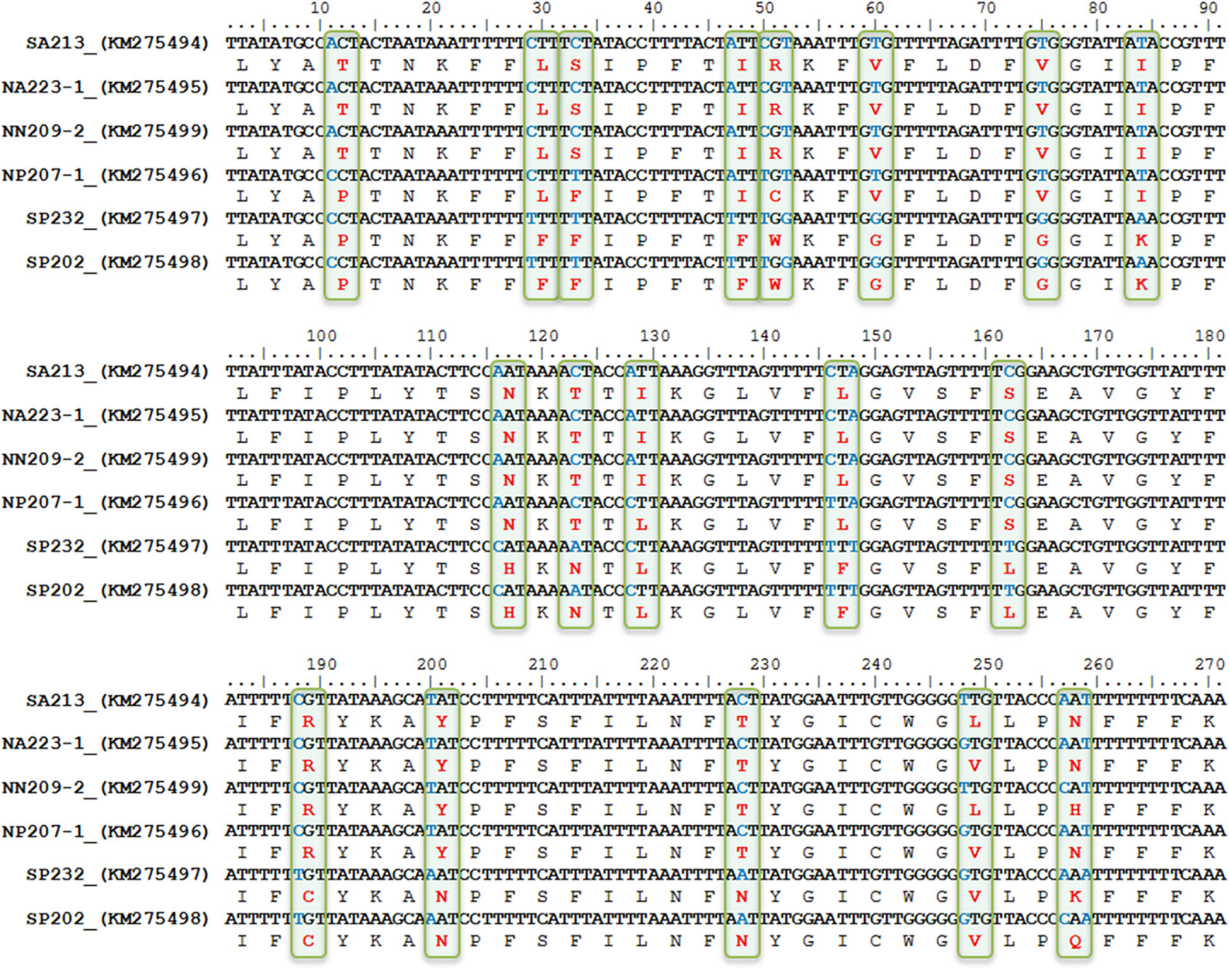
Single nucleotide polymorphisms (SNPs) and amino acid variations identified in *inmp* gene sequence of ‘*Ca*. P. phoenicium’ strains. Codons including SNPs are evidenced by green boxes. SNPs are shown in blue blod characters; amino acid variations are shown in red bold characters

Due to the role of phytoplasma membrane proteins in determining the vectoring activity of insects [36, 54] and the interaction with plant hosts [55], it is notable that within an extremely homogeneous population of AlmWB phytoplasma strains, the sole differences were revealed on integral membrane protein gene sequences. Alignments of nucleotide (358 nt) and amino acid (119 aa) sequences of *inmp* gene evidenced the presence of 21 nucleotide substitutions and all of them were non-synonymous (Fig. 3). Codon-based test of positive selection (dN/dS) carried out using different methods rejected the null hypothesis of strict-neutrality (d_N_ = d_S_) in favor of the alternative hypothesis (d_N_ > d_S_) (Table 3), showing the presence of a positive selection. Intriguingly, these preliminary data seem to indicate that AlmWB phytoplasma strains identified in peach plants are distinct from strains infecting almond and nectarine based on molecular markers within the *inmp* nucleotide sequences. Recently, insect species of the families *Cicadellidae* and *Cixiidae* have been reported to be capable of transmitting AlmWB phytoplasma [56, 57]. Considering this evidence, it is reasonable to hypothesize the possible implication of *inmp* diversity on multiple vector-specific epidemiological cycles of AlmWB phytoplasma in the diverse plant hosts. As for other phytoplasmas associated with important diseases (i.e., grapevine Flavescence dorée) [58, 59], the high genetic homogeneity within ‘*Ca*. P. phoenicium’ strains suggests that originally a unique strain (or a few strains) entered Lebanon, where variety in ecological niches lead to the clonal replication of this strain and its further adaptation to diverse environments, i.e. by relatively frequent mutation of the nucleotide and amino acid sequences of the membrane proteins interacting with hosts, as reported for ‘*Ca*. P. asteris’ strain OY-M and ‘*Ca*. P. solani’ [36, 60]. Molecular markers identified on the *inmp* gene in the present study will be employed for multiple gene sequence analyses of AlmWB phytoplasma strains identified in plant crops, insect vectors and weeds hosting the vectors, in order to increase knowledge of AlmWB disease spread and to improve possibilities for the development of sustainable strategies for its management.

**Table 3:** Codon-based test of positive selection for analysis between *inmp* sequences of ‘*Ca*. P. phoenicium’ strains

Model	Prob^a^	Stat^b^
Modified Nei-Gojobori Method (Proportion)	0.026	1.961
Modified Nei-Gojobori Method (Jukes-Cantor)	0.034	1.836
Pamilo-Bianchi-Li Method (Kimura 2-para)	0.015	2.191
Kumar Method (Kimura 2-para)	0.015	2.195

^a^ The probability of rejecting the null hypothesis of strict-neutrality (d_N_ = d_S_) in favor of the alternative hypothesis (d_N_ > d_S_). Values of *P* less than 0.05 are considered significant at the 5 % level

^b^ The test statistic (d_N_ - d_S_) is shown. d_S_ and d_N_ are the numbers of synonymous and nonsynonymous substitutions per site, respectively. The variance of the difference was computed using the bootstrap method (1000 replicates)

### Identification of phytoplasma-host interaction key genes within ‘*Ca*. P. phoenicium’ draft genome

Phobius prediction analyses of transmembrane (TM) domains and signal peptides (SPs) within annotated protein sequences of ‘*Ca*. P. phoenicium’ draft genome resulted in the assignment of 247 cytosolic proteins (not exhibiting a TM domain or a SP), 69 membrane proteins (characterized by at least one TM domain), three cell surface proteins (characterized by SP and TM domain(s)), and 14 secreted proteins (characterized by a SP and no TM domain) (Additional file 1: Table S1). Recent studies demonstrated that phytoplasma membrane proteins, exposing extracellular domain(s)/loop(s) within the host cell cytoplasm, and proteins secreted to host cell cytoplasm play a crucial role in the interactions between phytoplasmas and hosts, determining respectively the insect vectoring activity and specificity and the re-programming of gene expression in plant hosts [54, 61–65].

**Fig. 4:**
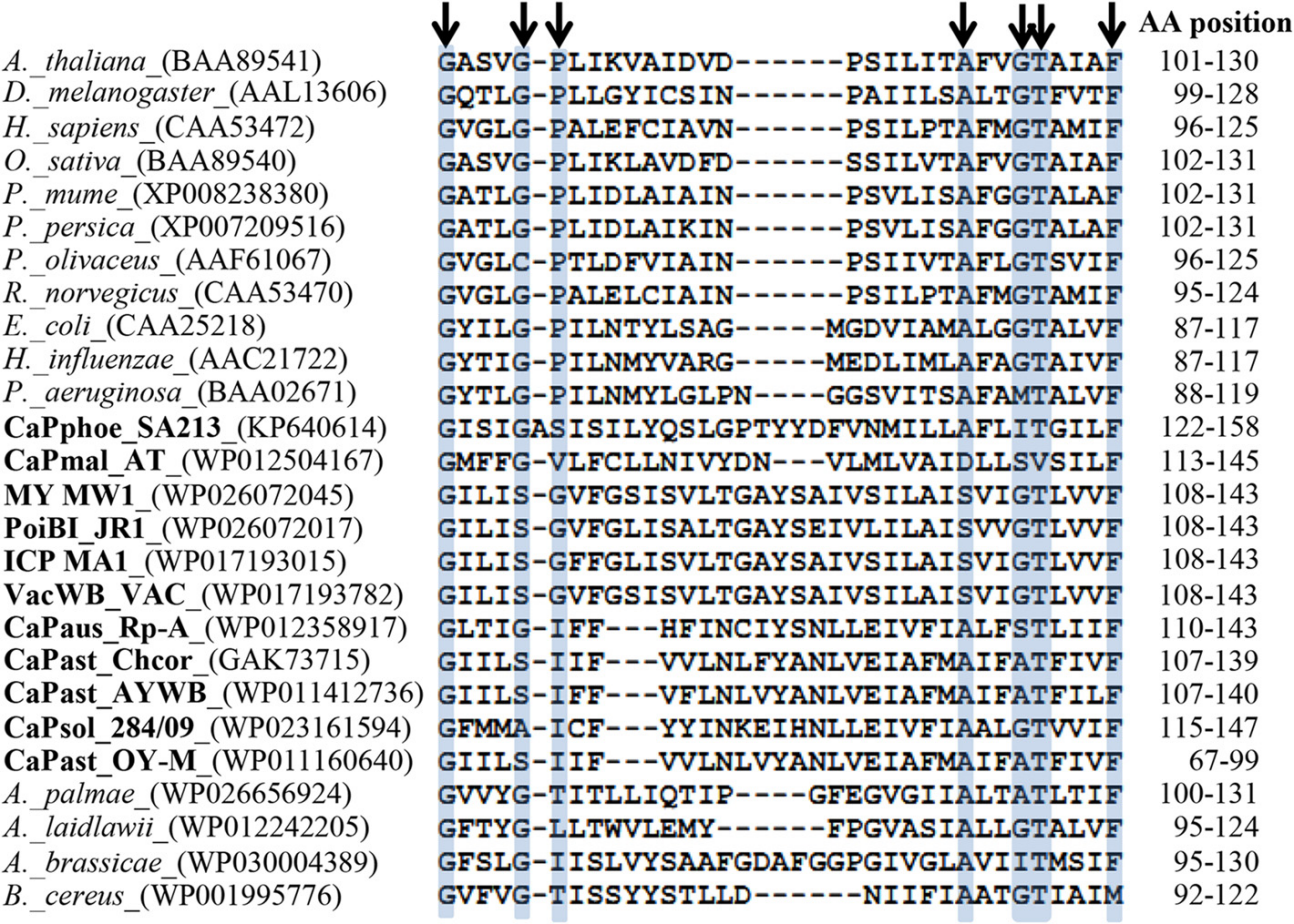
BI-1 amino acid patterns. BI-1 protein sequences of phytoplasmas, acholeplasmas, bacteria, plants and animals were retrieved from NCBI GenBank. Acronyms of phytoplasmas are in bold characters. Black arrows indicate the amino acid positions in the conserved BI-1 trans-kingdom pattern. Amino acid pattern starts in the third of seven predicted transmembrane domains and ends in the fourth transmembrane domain. Pattern positions are indicated in the column named AA position

**Fig. 5:**
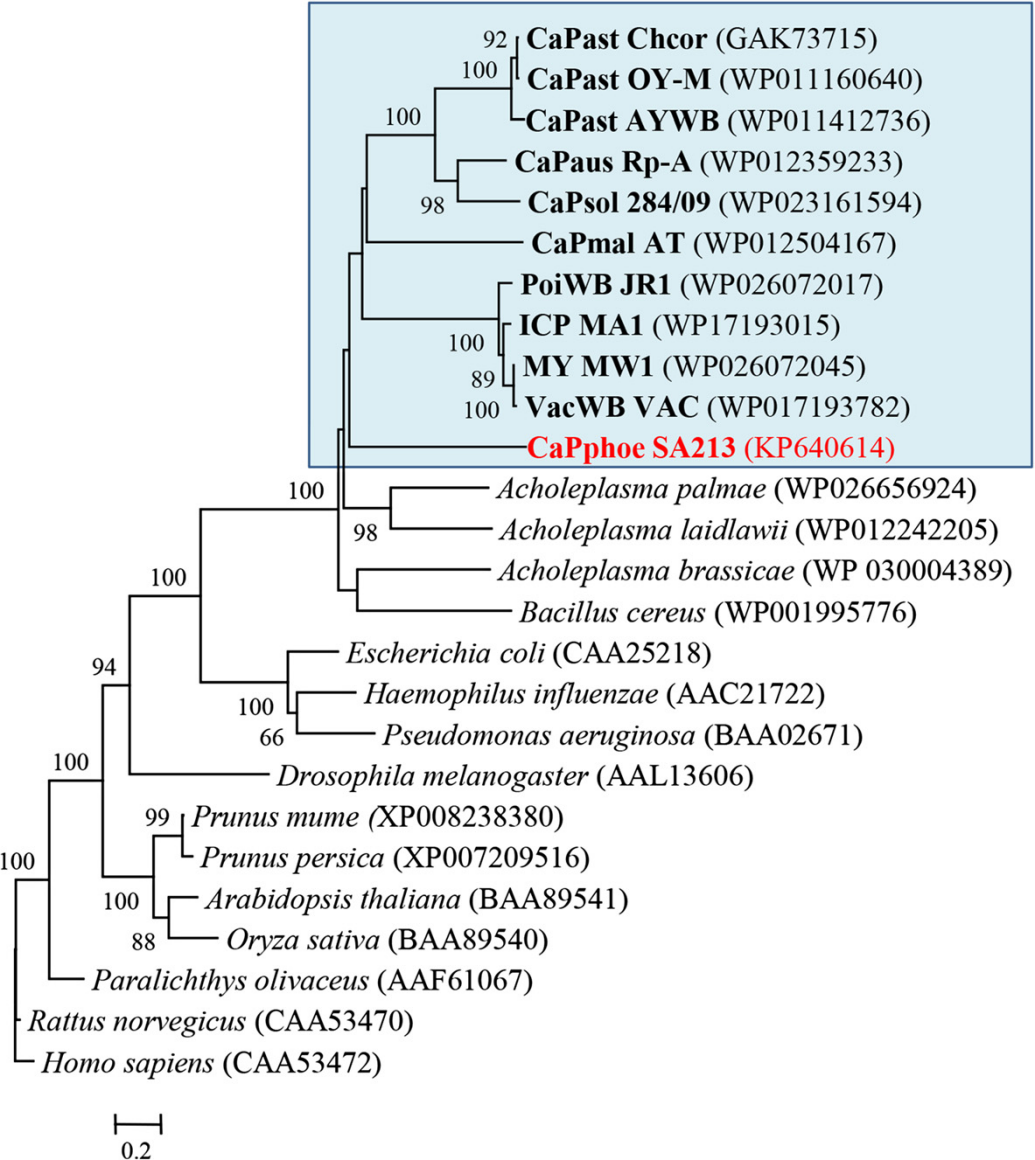
Phylogenetic tree inferred from BI-1 amino acid sequences. Minimum evolution analysis was carried out using the neighbor-joining method and bootstrap replicated 1,000 times. BI-1 protein sequences of phytoplasmas, acholeplasmas, bacteria, plants and animals were retrieved from NCBI GenBank. The GenBank accession number of each sequence is given in parenthesis. Phytoplasma strains (acronyms are in the legend of Fig. 1) are in bold characters; AlmWB phytoplasma strain SA213, identified in the present study, is in red bold characters

One of those integral membrane proteins (AlmWB_00860) was predicted as Bax inhibitor-1 (BI-1), an inhibitor of apoptosis-promoting Bax factor (sequence homology score: 25-31 % in comparison with phytoplasmas and prokaryotes; < 10 % in comparison with eukaryotic organisms). This anti-apoptotic gene has usually been identified within the genomes of plants and animals, but has also been reported for prokaryotes [66, 67], and is known to induce a reduction of programmed cell death (PCD) [68]. It remains remarkable that the overexpression of Bax inhibitor-1 in plants decreases resistance to pathogens [69, 70]. *BI-1* nucleotide sequences of AlmWB phytoplasma strains analyzed in the present study were identical to the sequence of the strain SA213 (data not shown), deposited in NCBI GenBank at accession number KP640614. BlastP and InterProScan 4 analyses revealed the presence of putative genes coding for inhibitors of Bax factor, originally assigned hypothetical proteins, in the draft and complete phytoplasma genome sequences. Alignment of BI-1 amino acid sequences of phytoplasmas, acholeplasmas, prokaryotes, plants and animals evidenced the presence of conserved trans-kingdom amino acid patterns (Fig. 4) determining the architecture of the protein [68] (Table 4). Moreover, BI-1 based phylogenetic clustering (Fig. 5) is consistent with that obtained by analyzing housekeeping genes, supporting the idea that *BI-1* is an evolutionary conserved gene and might be an ancient PCD regulator of general importance for cellular homeostasis. It is intriguing to discuss the possible role of BI-1, positioned in membrane protein, in phytoplasma interaction with hosts. In plants, BI-1 is an endoplasmic reticulum (ER)-resident transmembrane protein that can interact with multiple partners to alter intracellular Ca^2+^ flux control and lipid dynamics. Functionally, the level of BI-1 protein has been hypothesized to have the role of a rheostat to regulate the threshold of ER-stress inducible cell death [71]. It is largely reported that phytoplasmas can colonize not only phloem sieve elements, but also companion cells [72]. Further studies should be carried out to investigate the activity of phytoplasma BI-1 inside host(s). In particular, it should be interesting to verify if, inside companion cells, phytoplasma BI-1 could act in synergy with plant ER-resident BI-1 influencing the Ca^2+^ homeostasis and determining the signals related with PCD. In this way, BI-1 may act as a phytoplasma fitness-increasing factor by modifying the defense response of host plants. Alternatively, BI-1 could have a internal function in homeostasis and lipid dynamics inside phytoplasma cells.

**Table 4:** Phobius prediction of BI-1 protein domains in organisms of diverse kingdoms

BI-1 amino acid position			Phobius prediction
*Arabidopsis thaliana*	*Escherichia coli*	'*Ca*. P. phoenicium' SA213	
BAA89541	CAA25218	KP640614	
1–38	1–19	1–51	Cytoplasmic
39–56	20–41	52–72	Transmembrane
57–61	42–46	73–77	Non cytoplasmic
62–80	47–67	78–98	Transmembrane
81–91	68–73	99–109	Cytoplasmic
92–112	74–94	110–131	Transmembrane
113–117	95–105	132–142	Non cytoplasmic
118–139	106–126	143–165	Transmembrane
140–145	127–132	166–176	Cytoplasmic
146–169	133–154	177–199	Transmembrane
170–174	155–159	200–204	Non cytoplasmic
175–193	160–184	205–223	Transmembrane
194–213	185–195	224–243	Cytoplasmic
214–232	196–216	244–263	Transmembrane
233–247	217–219	264–275	Non cytoplasmic

Two putative secreted effector proteins, containing sequence-variable mosaic (SVM) sequence (SAP05- and SAP11-like), and one predicted hemolysin, containing a cystathionine-beta-synthase (CBS) domain, were identified within the predicted secreted proteins. Effectors have been described as small proteins, positioned within potential mobile units and/or phage-related SVM genomic regions of phytoplasma genomes, able to exit the phloem (where phytoplasmas are confined), spread to plant tissues, and interact directly or indirectly with plant nuclear transcription factors inducing or repressing the expression of specific plant genes [61–63]. Effector searching among phytoplasma genomes revealed the presence of SAP11-like proteins in AlmWB phytoplasma draft genome and in the genomes of ‘*Ca*. P. asteris’, ‘*Ca*. P. mali’, ‘*Ca*. P. australiense’, and PnWB phytoplasma (data not shown). Furthermore, genes coding for other phage-related elements were identified within the AlmWB phytoplasma draft genome, such as phage integrase (AlmWB_00650, AlmWB_02430, AlmWB_02490), and YqaJ-like viral recombinase (AlmWB_00050, AlmWB_00770).

### Further features from ‘*Ca*. P. phoenicium’ draft genome

Common gene sets for phytoplasmas encoding proteins for replication, DNA modification and structure, and DNA repair [73] were identified. The AlmWB phytoplasma draft genome included the entire S10-*spc*-alpha superoperon not encoding the gene *adk*, showing a *spc* operon with a gene order *rplO*-*secY*-*map*. Such gene organization within S10-*spc*-alpha superoperon is present also in ‘*Ca*. P. mali’ and PnWB phytoplasma group (‘*Ca*. P. australasia’), and diverges from that reported in ‘*Ca*. P. asteris’, ‘*Ca*. P. australiense’, ‘*Ca*. P. solani’ and *Acholeplasma* sp. [74]. Also the streptomycin (*str*) operon, carrying the genes *rpsL*, *rpsG*, *fusA*, and *tufB*, was completely identified in the AlmWB phytoplasma draft genome.

Common ABC-transporters for the ATP-dependent putative import of manganese/zinc, cobalt, spermidine/putrescine, methionine, oligopeptide and glycerol-3-phosphate were encoded in the draft genome sequence analyzed here. Furthermore, an ABC-transporter for the ATP-dependent multidrug resistance with permease function was identified in the AlmWB phytoplasma draft genome. A gene set necessary for building the *Sec*-dependent pathway was identified (*ffh*, *ftsH*, *secA*, *secY*, *yidC*) except the gene *secE*. As already shown for the ‘*Ca*. P. mali’ genome, the genes *groEL* and *groES*, coding for heat shock protein 60 family chaperones, were not in the sequence synteny *groEL*-*amp*-*nadE* common to other phytoplasmas [36].

With regard to the carbohydrate metabolism, the suggested conserved ATP-providing pathway of phytoplasmas has been identified encoded in all complete five phytoplasma genomes and in the draft sequences of AlmWB and PnWB phytoplasmas (this study, [73]). This pathway depends on the uptake of carboxylic acids (such as malate or oxaloacetate) mediated by the symporter MleP in phytoplasmas [65]. The draft sequence of PnWB phytoplasma also highlights the genetic repertoire for the utilization of citrate, which can be also imported by MleP (*syn.* CitS). The citrate lyase complex (CytEF) mediates the formation of oxaloacetate and acetate from citrate. Oxaloacetate may be decarboxylated by the common malate dehydrogenases encoded in phytoplasmas [65]. This central enzyme and MleP indicate a peculiarity of the phytoplasmas separating them from the genus *Acholeplasma* [75] and supporting their phylogenetic Gram-positive origin [65]. The utilization of oxaloacetate, beside malate, requires a malate dehydrogenase decarboxylating both substrates to produce pyruvate [65]. This step might be performed by NAD(P)H dependent malate dehydrogenase. *In silico* analysis of phytoplasmal malate dehydrogenases enabled the identification of the NADP^+^ binding motif (IPR016040) indicating a MaeB-like malate dehydrogenase (EC 1.1.1.38 and EC 1.1.1.40). The generated pyruvate is the entry molecule of the encoded pyruvate dehydrogenase multienzyme complex (PdhABCD) enabling the formation of acetyl-CoA, which is subsequently converted to acetyl-phosphate by the PduL-like phospotransacetylase and metabolized to acetate (AckA) releasing ATP [27, 73]. Experimental verification of the functions fulfilled by the key enzymes malate dehydrogenase (SfcA/MaeB) and phosphotransacetylase (PduL) was provided recently for ‘*Ca*. P. asteris’ [76].

## Conclusion

In the present study, we first determined the draft genome sequence of ‘*Ca*. P. phoenicium’ associated with AlmWB disease in Lebanon. The results obtained revealed important insights into the genetic diversity among ‘*Ca*. P. phoenicium’ strains associated with the disease. Multiple gene typing analyses of ‘*Ca*. P. phoenicium’ strains infecting almond, peach and nectarine in Lebanon (i) revealed a substantial genetic homogeneity within the analyzed phytoplasma populations based on house-keeping gene sequence analyses, and (ii) allowed the identification of distinct AlmWB-associated phytoplasma strains from diverse host plants based on *inmp* (integral membrane protein) gene sequence analysis. This evidence, along with prior reports of multiple insect vectors of AlmWB phytoplasma [56, 57], suggests that AlmWB could be associated with phytoplasma strains derived from the adaptation of an original strain to diverse hosts.

Analyses of the available genome features allowed the identification of candidate determinants of pathogenicity and highlighted the coding of the conserved ATP-providing pathway of phytoplasmas, based on MleP/CitS mediated malate uptake and subsequent formation of acetate. Two putative secreted effector proteins, containing SVM sequence (SAP05- and SAP11-like), and one predicted hemolysin, containing a CBS domain, were identified within the predicted secreted proteins and can be proposed as pathogenicity determinants. Intriguingly, the identification of a putative inhibitor of apoptosis-promoting Bax factor in AlmWB phytoplasma draft genome and within genomes of other ‘*Ca*. Phytoplasma’ species suggested its potential role as a phytoplasma fitness-increasing factor by modification of the host-defense response. Reports of reduction of leaf cell death in phytoplasma-infected plants [77], and increased fitness of phytoplasma-infected insect vectors [78, 79] could support this hypothesis.

## Additional files

Additional file 1: Table S1. Annotated genes within the draft genome of ‘*Ca*. P. phoenicium’ strain SA213. (XLS 85 kb) Additional file 2: Table S2. PanOCT analysis of proteins identified within ‘*Ca*. P. phoenicium’ strain SA213 draft genome and phytoplasma core genome. (XLS 115 kb) Additional file 3: Table S3. Tblastn comparison of ‘*Ca*. P. phoenicium’ strain SA213 draft genome with other phytoplasmas. (XLS 103 kb) Additional file 4: Table S4.
*Inmp* gene nucleotide sequence identity matrix within ‘*Ca*. P. phoenicium’ strains. (XLS 18 kb)
